# Projected Prevalence and Economic Burden of Alzheimer’s Disease and Related Dementias in China: Regional Disparities and Policy Implications

**DOI:** 10.34133/hds.0377

**Published:** 2025-11-21

**Authors:** Xinyi Liu, Simiao Chen, Donglan Zhang, Yian Gu, Gang Li, Bei Wu, José A. Pagán, Luxia Zhang, Yan Li

**Affiliations:** ^1^ School of Public Health, Shanghai Jiao Tong University School of Medicine, Shanghai, China.; ^2^ School of Population Medicine and Public Health, Chinese Academy of Medical Sciences and Peking Union Medical College, Beijing, China.; ^3^ Heidelberg Institute of Global Health, Faculty of Medicine and University Hospital, Heidelberg University, Heidelberg, Germany.; ^4^ State Key Laboratory of Respiratory Health and Multimorbidity, Beijing, China.; ^5^ Center for Population Health and Health Services Research, Department of Foundations of Medicine, New York University Grossman Long Island School of Medicine, Mineola, NY, USA.; ^6^ Department of Population Health, New York University Grossman School of Medicine, New York, NY, USA.; ^7^ Department of Epidemiology, Joseph P. Mailman School of Public Health, Columbia University, New York, NY, USA.; ^8^ Taub Institute for Research on Alzheimer’s Disease and the Aging Brain, Gertrude H. Sergievsky Center, and Department of Neurology, Columbia University, New York, NY, USA.; ^9^ Rory Meyers College of Nursing, New York University, New York, NY, USA.; ^10^ Department of Public Health Policy and Management, School of Global Public Health, New York University, New York, NY, USA.; ^11^ National Institute of Health Data Science at Peking University, Beijing, China.; ^12^ Renal Division, Department of Medicine, Peking University First Hospital, Peking University Institute of Nephrology, State Key Laboratory of Vascular Homeostasis and Remodeling, Peking University, Beijing, China.; ^13^ Center for Digital Health and Artificial Intelligence, Peking University First Hospital, Beijing, China.; ^14^ Advanced Institute of Information Technology, Peking University, Hangzhou, China.; ^15^ Department of Population Health Science and Policy, Icahn School of Medicine at Mount Sinai, New York, NY, USA.

## Abstract

**Background:** China has the largest population with Alzheimer’s disease and related dementias (ADRDs) globally, and rapid population aging is expected to drive a substantial increase in cases. This study projects ADRD prevalence and associated economic burdens across provinces in China from 2025 to 2060. **Methods:** Using data from the China Health and Retirement Longitudinal Study (CHARLS) supplemented by national demographic and provincial statistics, we projected the prevalence and care costs of ADRD for each of the 31 provinces in China from 2025 to 2060. Cost projections included formal care expenses and informal caregiving valued through replacement cost methods. We conducted uncertainty analysis to provide robust estimates for ADRD prevalence and costs. **Results:** By 2060, ADRD cases in China are projected to reach approximately 49.89 million, with the highest prevalence and economic burden concentrated in provinces such as Shandong, Sichuan, Jiangsu, Henan, and Guangdong. Formal care costs alone are expected to exceed $1 trillion annually, while the total economic value—including informal caregiving—could surpass $5 trillion. Geographic disparities highlight that Eastern and Central regions, with a higher proportions of older adults, will bear disproportionate costs. Informal caregiving is projected to constitute 60% to 80% of total ADRD-related costs. **Conclusion:** China faces an unprecedented rise in ADRD-related economic burden over the next 4 decades, with substantial regional disparities. Strengthening long-term care infrastructure, expanding financial and social support for caregivers, and implementing regionally tailored healthy aging policies are essential to ensuring equitable and sustainable ADRD care across China.

## Introduction

China is experiencing a profound demographic shift driven by rapid population aging. By 2050, the proportion of individuals aged 65 years and older is projected to more than double, reaching approximately 366 million, or 26% of the total population [1]. This demographic change will likely lead to a remarkable rise in age-related diseases such as the Alzheimer’s disease and related dementias (ADRDs) [2]. Current estimates suggest that around 15 million people in China are living with ADRDs [3], and this number is anticipated to increase dramatically in the coming decades as the aging trend continues. In China, Alzheimer’s disease alone has become the fifth-leading cause of death [3]. Between 1990 and 2019, the disability-adjusted life years associated with ADRDs increased by 225.7% [4], reflecting the growing impact on both affected individuals, their families, and the healthcare system [3].

As the prevalence of ADRDs increases, the economic burden associated with these conditions is expected to increase considerably. Previous studies suggest that the social and economic costs of dementia—encompassing direct healthcare expenses, social care expenditures, and the costs of informal caregiving—will grow substantially [2,5]. As more individuals require comprehensive care in formal settings such as hospitals and nursing homes, as well as community and informal family support [2], the financial strain on families, China’s healthcare system, and the economy will intensify. Yet, few studies have projected the prevalence and economic burden of ADRDs in China. This knowledge gap is further complicated by the regional disparities within China. Differences in economic development, healthcare infrastructure, and demographic composition across provinces may lead to notable variation in ADRD prevalence rates and care costs [6].

This study aims to provide detailed projections of ADRDs prevalence and associated care costs across different provinces in China, from 2025 to 2060. By integrating national and provincial data, this study offers valuable insights into the future burden of ADRDs in China. Understanding the province-level dynamics of ADRDs will also be essential for developing effective, region-specific public health strategies.

## Methods

### Data sources

To estimate ADRD prevalence, we used data from the China Health and Retirement Longitudinal Study (CHARLS), a nationally representative longitudinal survey designed to collect high-quality data on individuals aged 45 and older, as well as their families, across China. The baseline survey, conducted in 2011, covered 150 county-level units and 450 village-level units, sampling approximately 17,000 individuals from around 10,000 households. Follow-up surveys have been conducted every 2 to 3 years. For this study, we used data from the first 4 waves of CHARLS (2011, 2013, 2015, and 2018). The 2020 wave was excluded because it lacks individual-level outpatient and inpatient medical expenditure variables required for cost modeling, and healthcare utilization during 2020 was atypically affected by the COVID-19 pandemic.

In addition, we incorporated data from multiple sources to estimate ADRD care costs across provinces. Specifically, we used the China Statistical Yearbook to obtain data of total population, provincial population, and the Consumer Price Index (CPI). From the Provincial Statistical Yearbooks, we extracted data on the average wages in the healthcare industry and service industry to estimate informal care costs of ADRDs across provinces. Additionally, we utilized population projection data from the World Population Prospects of the United Nations, which provided estimates of China’s population growth from 2025 to 2060. Table S1 provides a detailed summary of all data sources used in this study.

### Cognitive and functional assessment in CHARLS

ADRDs in this study were defined by the presence of both cognitive impairment (CI) and functional impairment (FI) or the documented use of medications for treating memory-related conditions [6,7]. CI was determined based on deficits in at least 2 of 3 cognitive domains, including time orientation, memory (immediate or delayed recall), and arithmetic skills [6]. The assessment was conducted using data from the CHARLS dataset based on a modified version of the Telephone Interview of Cognitive Status. Because educational attainment has a substantial influence on cognitive test performance independent of underlying cognitive status, we standardized the thresholds for impairment within education strata. Individuals with lower educational attainment typically perform less well on tests such as word recall or serial subtraction despite comparable cognitive ability, which may lead to overestimation of impairment if a single cutoff is applied to all participants. To minimize such misclassification, we followed the CHARLS convention and prior population-based studies by calculating impairment thresholds (≥1.5 SD below the mean) separately within 4 education categories: no formal education, primary school, middle school, and high school or above. This approach provides a more accurate identification of CI across heterogeneous educational backgrounds [6,7].

The cognitive assessments and their scoring have been extensively described and used in CHARLS and other related aging studies [8–10]. A recent study based on the CHARLS Harmonized Cognitive Assessment Protocol further supports their feasibility and construct validity in Chinese populations [11]. Detailed item descriptions, scoring methods, and score ranges are provided in Table S2. FI was defined as the inability to independently perform one or more basic activities of daily living (BADLs), which include tasks such as bathing, dressing, toileting, eating, and getting in and out of bed [6,7]. The self-reported BADL was also from the CHARLS dataset.

Our case definition adopts a survey-based operational approach commonly used in large-scale population studies where clinical adjudication is unavailable [6,7,12]. It is worth noting that this algorithmic definition differs from a clinically confirmed diagnosis and may introduce some misclassification [13,14]. However, CHARLS is one of the largest nationally representative aging cohorts in China, featuring harmonized cognitive and functional assessments and rigorous sampling [15,16]. Leveraging this dataset enables credible population-level estimation of ADRD burden in the absence of standardized national diagnostic systems. Accordingly, our projections should be interpreted as survey-based, policy-relevant estimates derived from a representative sample.

### Estimation of individuals’ ADRD probability

We applied a probit regression model to predict the likelihood of ADRD for each respondent, using CHARLS data from 2011 to 2018. Individual-level covariates include age, sex, education level, limitations in ADL (e.g., eating and bathing), limitations in IADL (e.g., managing money and preparing meals), and cognitive function scores. The model also accounted for changes in ADL limitations, IADL limitations, and cognitive scores from the 2 preceding CHARLS surveys.

Cognitive scores were assessed using a scoring method developed by the CHARLS team [17], where cognitive ability was calculated as the sum of scores for mental status and episodic memory. Mental status included tasks such as serial 7 subtraction, orientation questions (e.g., date and season), and redrawing overlapping pentagons. Episodic memory was measured by averaging the scores from immediate and delayed recall of a list of 10 words. The resulting composite cognitive score ranged from 1 to 21, providing a reliable assessment of cognitive performance. Using the estimated coefficients from the probit model, we calculated the probability of ADRDs for all CHARLS respondents. Our approach for estimating ADRD probabilities is similar to those used by Hurd et al. [18] and Nandi et al. [2].

### Estimation of ADRD costs of care

Individuals with ADRDs typically exhibit more comorbidities than those without, which independently contributes to higher healthcare costs. To isolate the costs specifically attributable to ADRDs, we employed multivariate regression models, linking each cost component to the estimated probability of ADRDs while controlling for comorbidities and demographic factors. Comorbidities included hypertension, dyslipidemia, diabetes, cancer, chronic lung disease, liver disease, cardiovascular conditions (e.g., myocardial infarction, coronary heart disease, angina, congestive heart failure, and stroke), kidney disease, gastrointestinal disorders, psychiatric conditions, arthritis or rheumatism, and asthma. Demographic variables encompassed age, sex, ethnicity, education level, marital status, urban or rural residence, geographic region (eastern, central, western, or northeastern China), number of children, and annual per capita household consumption. The regression model used was:

$${\text{cost}}_{\mathrm{iy}}=\alpha +\beta_{1}P{\left(\text{ADRDs}\right)}_{\mathrm{iy}}+\beta_{2}X_{\mathrm{iy}}+u_{\text{iy}}$$ (1)

where cost*_iy_* denotes the cost of care for individual *i* in year *y* (2015 to 2018). Our analysis included individuals aged 50 years and above. The estimated probability of dementia, $P{\left(\text{ADRDs}\right)}_{\mathrm{iy}}$, was derived from the probit regression mentioned above, and *X* encompasses demographic factors and comorbidities. The coefficient $\beta_{1}$ was interpreted as the increase in costs associated with a shift in the probability of ADRDs from 0 to 1, controlling for comorbidities and demographic characteristics.

We calculated 2 types of care costs for ADRDs: the formal and informal care costs. The formal care costs included expenditures incurred in healthcare settings, including outpatient and inpatient medical expenses, out-of-pocket payments, and total medical costs (including both outpatient and inpatient medical expenses) reported by respondents. These costs were estimated based on self-reported healthcare utilization and expenses from the CHARLS survey. The informal care costs represented the economic value of unpaid care provided by family members or relatives, such as spouses, children, and other household members. We used the replacement cost method [2], valuing caregiving time at the market rate for equivalent services provided by professional agencies, based on self-reported caregiving hours per week or month. Average wages from the service and healthcare sectors were applied to calculate replacement costs, using data from Provincial Statistical Yearbooks to adjust for regional economic differences.

All cost estimates were standardized to 2018 values using the healthcare CPI from China’s statistical yearbooks and converted to 2018 US dollars based on the average exchange rate, enabling international comparisons. Standard errors were calculated through 2,000 bootstrap simulations, and the results are presented as estimated coefficients with 95% confidence intervals, reflecting the per-patient cost of ADRD care.

### Future projections

For future projections of ADRD care costs, we used 2021 provincial prevalence rates from the China Alzheimer’s Report 2024 [19]. To estimate future ADRD prevalence, we applied the average annual growth rate of global ADRD prevalence from 1990 to 2021 based on data from the Global Burden of Disease Study [20]. We assumed that ADRD prevalence across provinces in China would follow this trend and projected the number of ADRD patients from 2025 to 2060. We also conducted a one-way sensitivity analysis, varying the ADRD prevalence growth rate by ±30%, to assess its impact on projections of total cases and costs (Figs. S1 and S2).

These projections combined projected provincial prevalence rates with each province’s population proportion and population forecasts from the United Nations’ World Population Prospects [21]. This approach provided detailed estimates of ADRD patient numbers at 5-year intervals for each province from 2025 to 2060. Per-patient costs for both formal and informal care were assumed to increase by 3% annually [2].

To account for uncertainty in parameters in the projections, we conducted an uncertainty analysis. We allowed the annual growth rate of ADRD prevalence, healthcare cost inflation, and population projections to fluctuate randomly within a range of 75% to 125% of their baseline values. Using this joint uniform distribution, we generated 10,000 random samples to estimate the number of individuals with ADRD, formal care costs, and total costs (including informal care costs calculated via both methods) at 5-year intervals from 2025 to 2060. Results are presented as mean estimates with 95% confidence intervals, derived from these simulations, to capture the range of potential outcomes.

## Results

As Table 1 shows, the average predicted probability of ADRDs varied significantly across different demographic and health profiles. Specifically, males, older adults, individuals with lower educational attainment, and those who were divorced, separated, never married, or widowed showed a higher likelihood of having ADRDs. Additionally, individuals with one or more limitations in ADLs or IADLs were more likely to have ADRDs than individuals without these limitations. Moreover, the presence of comorbidities further heightened the likelihood of ADRDs, with all comorbidities—except liver disease—being significantly associated with increased risks. Table S3 summarizes the basic characteristics of respondents aged 50 and above from the CHARLS dataset, while Tables S4 and S5 present the probit regression results and model fit diagnostics for the ADRD prediction model.

**Table 1.** Probability of ADRDs according to the characteristics of the study population. Data are based on a total of 23,891 person-years. For each characteristic, such as gender and marital status, the probability of ADRDs was calculated from the regression of the predicted probability of ADRDs on indicator variables for the categories taken by that characteristic, such as “male” and “female” in the case of gender and “unmarried” and “married” in the case of marital status. *P* values indicate the null hypothesis that the ADRD probabilities are equivalent to those of the reference group or among different subgroups. CI denotes confidence interval.

Characteristic	Distribution percent (%)	Probability of dementia			*P* value for comparison
		Mean	95% CI		
Gender					
Female	50.78	0.024	0.023	0.025	0.002
Male	49.22	0.027	0.026	0.029	
Age					
50–54 years	16.76	0.011	0.010	0.011	0.089
55–59 years	18.50	0.019	0.017	0.020	
60–64 years	15.37	0.033	0.030	0.035	
65–69 years	17.36	0.047	0.043	0.051	
70–74 years	12.89	0.058	0.052	0.064	
75–79 years	8.97	0.051	0.042	0.059	
80–84 years	5.49	0.048	0.036	0.060	
85–89 years	2.74	0.064	0.017	0.144	
≥90 years	1.92	0.058	0.017	0.100	
Ethnicities ^a^					
Han	92.22	0.025	0.024	0.026	0.387
Others	7.78	0.026	0.022	0.029	
Educational level ^b^					
No formal education	39.61	0.025	0.024	0.027	0.003
Primary school	23.24	0.030	0.028	0.033	
Middle school	22.45	0.023	0.021	0.024	
High school or above	14.71	0.023	0.021	0.025	
Marital status ^c^					
Married	88.35	0.025	0.024	0.026	0.003
Separated/Divorced/Widowed/Never married	11.65	0.034	0.030	0.038	
Household registration ^d^					
Urban	24.03	0.026	0.024	0.028	0.726
Rural	75.97	0.026	0.025	0.027	
Limitations in ADLs ^e^					
No	61.77	0.021	0.020	0.022	<0.001
Yes	38.23	0.056	0.052	0.061	
Limitations in IADLs ^f^					
No	56.53	0.021	0.020	0.022	<0.001
Yes	43.47	0.050	0.046	0.054	
Coexisting conditions					
Hypertension ^g^	44.66	0.037	0.034	0.039	<0.001
Dyslipidemia ^h^	28.99	0.033	0.030	0.037	<0.001
Diabetes or high blood sugar ^i^	16.93	0.037	0.033	0.042	<0.001
Cancer ^j^	3.54	0.035	0.022	0.048	0.044
Chronic lung diseases ^k^	20.41	0.038	0.034	0.042	<0.001
Liver disease ^l^	9.74	0.029	0.024	0.035	0.093
Heart attack, coronary heart disease, angina, congestive heart failure, or other heart problems ^m^	25.23	0.036	0.033	0.040	<0.001
Stroke ^n^	9.40	0.073	0.060	0.085	<0.001
Kidney disease ^o^	13.93	0.032	0.028	0.036	0.002
Stomach or other digestive disease ^p^	36.21	0.029	0.027	0.031	0.001
Emotional, nervous, or psychiatric problems ^q^	4.45	0.042	0.031	0.054	0.001
Arthritis or rheumatism ^r^	46.86	0.033	0.031	0.035	<0.001
Asthma ^s^	8.36	0.047	0.041	0.054	<0.001

^a^ With 690 missing values.

^b^ With 17 missing values.

^c^ With 4 missing values.

^d^ With 238 missing values.

^e^ With 450 missing values.

^f^ With 294 missing values.

^g^ With 621 missing values.

^h^ With 789 missing values.

^i^ With 671 missing values.

^j^ With 634 missing values.

^k^ With 627 missing values.

^l^ With 661 missing values.

^m^ With 643 missing values.

^n^ With 619 missing values.

^o^ With 652 missing values.

^p^ With 608 missing values.

^q^ With 635 missing values.

^r^ With 608 missing values.

^s^ With 626 missing values.

Table 2 presents annual per-person cost estimates for ADRDs (see Tables S6 to S14 for detailed results). The estimated total annual cost for formal care attributable to ADRDs was $14,819 (95% CI, $8,428 to $21,211) per person. When adjusted for comorbidities and demographic factors, the cost estimate decreased to $8,288 (95% CI, $4,838 to $11,738) per person. Among these adjusted costs, hospitalization accounted for the largest share, approximately $4,831 (95% CI, $2,332 to $7,329). The out-of-pocket expenditures made up around 55% of the total formal healthcare costs, or about $4,553 (95% CI, $2,575 to $6,532). The estimated informal care costs attributable to ADRDs were $18,860 (95% CI, $17,294 to $20,426) and $32,387 (95% CI, $29,552 to $35,221) based on the replacement cost approach using the service industry and healthcare industry wage standards, respectively. These estimates remained stable after controlling for potential confounding factors. Calculated using the replacement cost of the service industry, the estimated informal care cost attributable to ADRD was $17,048 (95% CI, $14,989 to $19,107) per person. When using the average wage in the healthcare industry, the informal care cost increased to $29,921 (95% CI, $26,204 to $33,638) per person. In addition, the estimated annual cost for both formal and informal care attributable to ADRDs, adjusted for comorbidities and demographic factors, was $25,153 (95% CI, $21,026 to $29,279) per person based on the replacement cost in the service industry, and $37,776 (95% CI, $32,434 to $43,118) per person when calculated using the replacement cost in the healthcare industry.

**Table 2.** Yearly cost per person attributed to ADRDs in China, in 2018 US dollars. The 95% confidence intervals for the estimates are shown in parentheses.

Variable	Yearly cost per person	
	Unadjusted	Adjusted for demographic characteristics and coexisting conditions
Formal care		
Outpatient care cost	8,234 (2,262–14,206)	3,497 (1,187–5,807)
Hospitalization cost	6,279 (4,457–8,102)	4,831 (2,332–7,329)
Total out-of-pocket spending	8,501 (4,300–12,701)	4,553 (2,575–6,532)
Total medical cost (A)	14,819 (8,428–21,211)	8,288 (4,838–11,738)
**Informal care**		
Caregiving time valued according to replacement cost of service industry (B)	18,860 (17,294–20,426)	17,048 (14,989–19,107)
Caregiving time valued according to replacement cost of healthcare industry (C)	32,387 (29,552–35,221)	29,921 (26,204–33,638)
**Grand total**		
Total formal care cost plus caregiving time valued according to replacement cost of service industry (D = A + B)	33,592 (26,698–40,485)	25,153 (21,026–29,279)
Total formal care cost plus caregiving time valued according to replacement cost of healthcare industry (E = A + C)	47,053 (39,682–54,423)	37,776 (32,434–43,118)

Table 3 presents future projections for the number of ADRDs patients and the economic burden (in 2018 US dollars) in China. The total annual cost for formal ADRDs care was estimated at $205.94 billion (95% CI, $157.37 to $256.36 billion) in 2025, projected to rise to $1.46 trillion (95% CI, $0.79 to $2.41 trillion) by 2060. The total cost for both formal and informal care, calculated using the replacement cost from the service industry, was projected to increase from $624.99 billion (95% CI, $477.61 to $778.03 billion) in 2025 to $4.45 trillion (95% CI, $2.39 to $7.33 trillion) by 2060. When calculated using the replacement cost in the healthcare industry, the total care cost was projected to increase from $938.64 billion (95% CI, $717.30 to $1,168.48 billion) in 2025 to $6.68 trillion (95% CI, $3.59 to $11.00 trillion) by 2060.

**Table 3.** Projected number of ADRD patients and economic burden in China from 2025 to 2060. The 95% confidence intervals for the estimates are shown in parentheses.

Year	Total number of ADRDs patients in China (millions)	Formal care cost (billions of 2018 US$)	Grand total cost (billions of 2018 US$)	
			Total formal care cost plus caregiving time valued according to replacement cost of service industry	Total formal care cost plus caregiving time valued according to replacement cost of healthcare industry
2025	20.20 (15.58–24.91)	205.94 (157.37–256.36)	624.99 (477.61–778.03)	938.64 (717.30–1,168.48)
2030	23.36 (17.78–29.34)	276.33 (205.61–355.08)	838.64 (624.01–1,077.61)	1,259.50 (937.16–1,618.41)
2035	26.92 (20.04–34.49)	369.79 (264.43–494.02)	1,122.25 (802.51–1,499.29)	1,685.45 (1,205.25–2,251.71)
2040	30.85 (22.47–40.56)	492.24 (335.97–679.50)	1,493.89 (1,019.62–2,062.19)	2,243.60 (1,531.32–3,097.10)
2045	35.33 (24.97–47.19)	656.07 (423.93–949.51)	1,991.10 (1,286.56–2,881.62)	2,990.33 (1,932.22–4,327.76)
2050	40.00 (27.49–54.92)	864.44 (531.29–1,306.80)	2,623.47 (1,612.40–3,965.98)	3,940.06 (2,421.57–5,956.30)
2055	44.86 (30.02–63.34)	1,129.39 (650.19–1,775.04)	3,427.55 (1,973.24–5,387.03)	5,147.66 (2,963.51–8,090.50)
2060	49.89 (32.31–71.62)	1,464.73 (787.26–2,414.27)	4,445.25 (2,389.24–7,326.98)	6,676.10 (3,588.28–11,004.02)

**Fig. 1.**
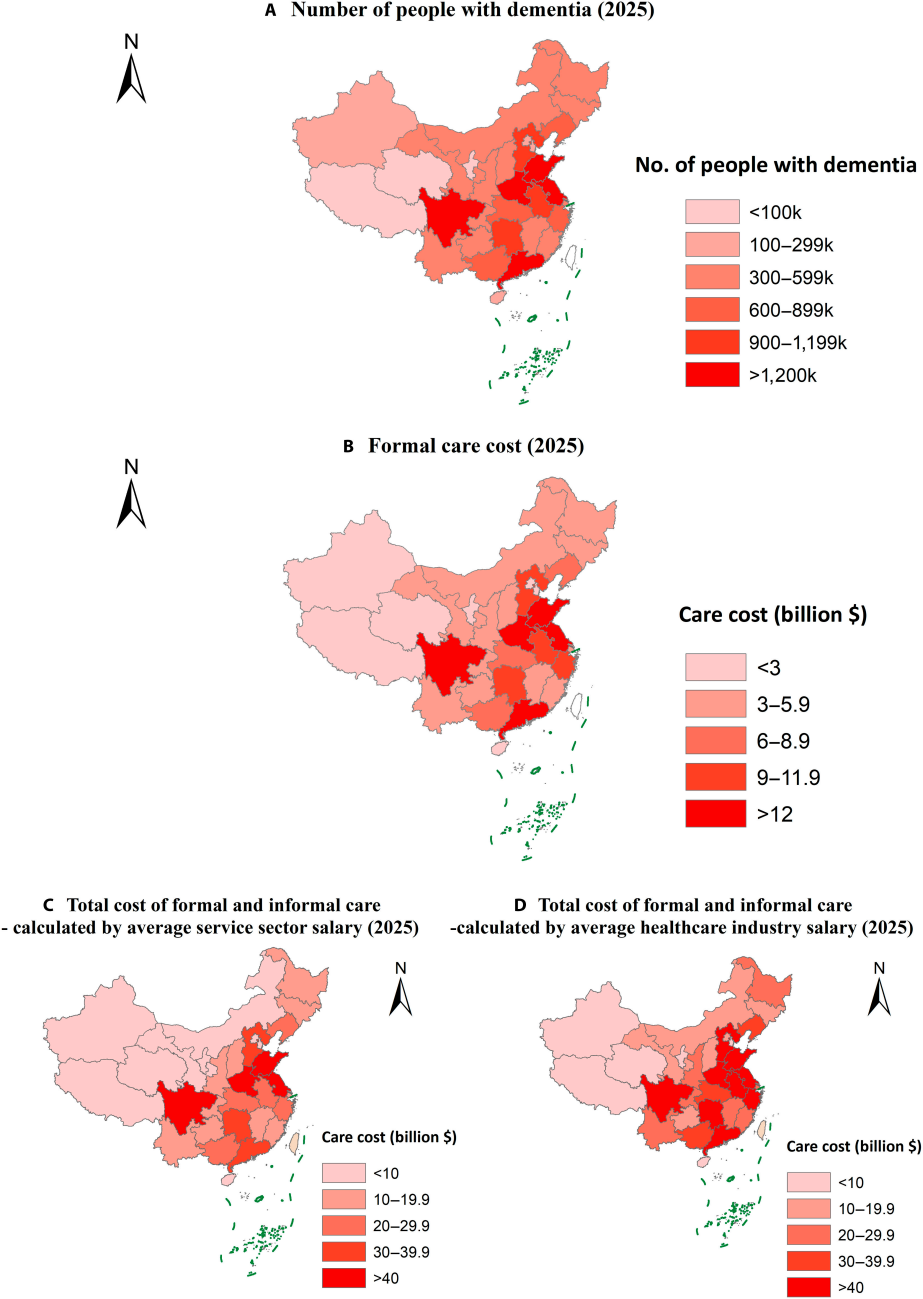
Projected number of ADRD patients and the associated economic burden in China, 2025 (in 2018 US dollars). (A) Number of people with dementia, 2025. (B) Formal care costs, 2025. (C) Total cost of formal and informal care—calculated by average service sector salary, 2025. (D) Total cost of formal and informal care—calculated by average healthcare industry salary, 2025.

**Fig. 2.**
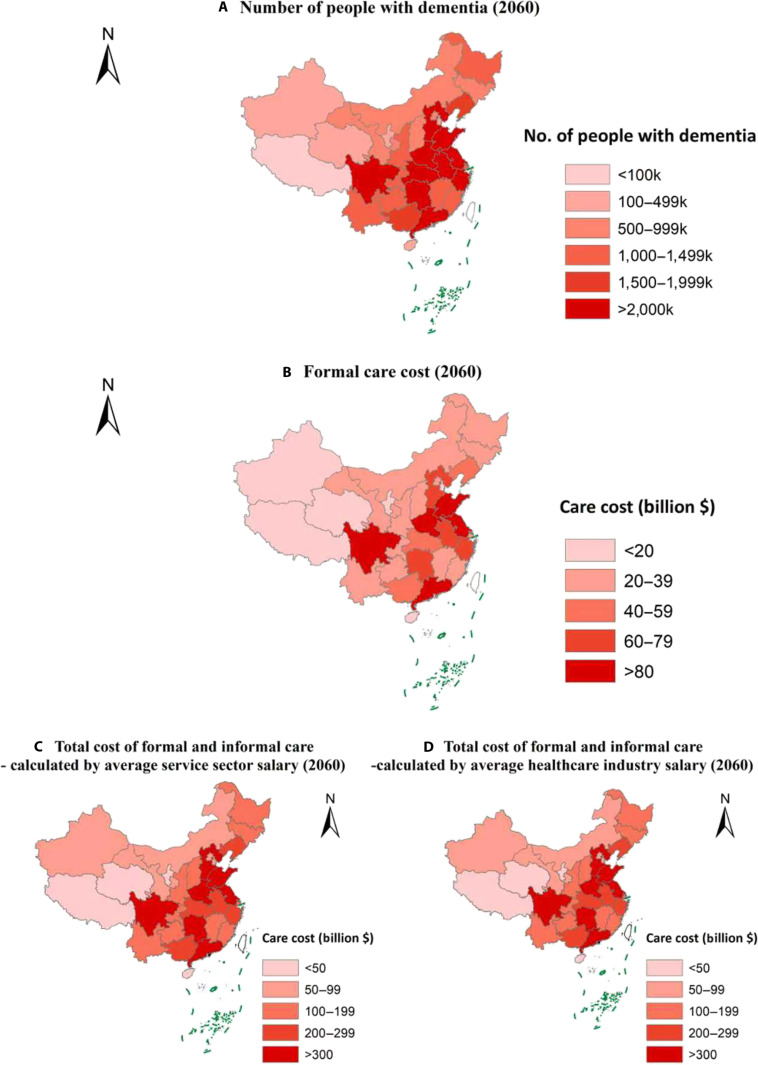
Projected number of ADRD patients and the associated economic burden in China, 2060 (in 2018 US dollars). (A) Number of people with dementia, 2060. (B) Formal care costs, 2060. (C) Total cost of formal and informal care—calculated by average service sector salary, 2060. (D) Total cost of formal and informal care—calculated by average healthcare industry salary, 2060.

Figures 1 and 2 depict the projected number of ADRD patients and associated economic burden (in 2018 US dollars) across provinces in China in 2025 and 2060 (see Tables S15 and S16 for detailed results). In both years, the prevalence of ADRDs was expected to be highest in the eastern and central regions of China. By 2060, provinces such as Shandong, Sichuan, Jiangsu, Henan, and Guangdong were projected to incur formal care costs for ADRDs exceeding $80 billion, with the total care cost (including both formal and informal care) anticipated to surpass $200 billion.

## Discussion

This study projects a substantial increase in both the prevalence and economic burden of ADRDs in China from 2025 to 2060. Findings suggest that by 2060, approximately 49.89 million individuals in China could be living with ADRDs, with formal care costs surpassing $1 trillion annually and total costs—including both formal and informal care—approaching $5 trillion annually. This projected growing burden highlights an urgent need for targeted healthcare policies—including long-term care growth [22]—and resource allocation strategies, particularly in regions with vulnerable aging populations, less developed economies, and disparities in healthcare and long-term care infrastructure.

Our projections align with global trends, which also indicate a dramatic rise in ADRD prevalence and associated economic burden [23]. The World Health Organization estimates that the global population living with dementia is currently around 55.2 million, expected to reach 139 million by 2050 [23]. Similarly, Alzheimer’s Disease International (ADI) projects the global cost of dementia to increase from $1.3 trillion in 2020 to $2.8 trillion by 2030 [24]. Our findings indicate that China will contribute substantially to this global economic burden due to its rapid population aging and increasing life expectancy [23]. While the prevalence of ADRDs is rising globally, regional variations exist. Developed countries, such as the United States, have faced substantial economic impact; in 2020 alone, the United States incurred $196 billion in direct medical costs for ADRDs, with an additional $450 billion attributed to informal caregiving [2]. In contrast, developing countries, including China, are experiencing a more rapid increase in ADRD prevalence driven by demographic shifts [25], resulting in an economic burden that requires more effective and tailored healthcare and public health interventions.

Our projections are consistent with previous studies forecasting an increase in ADRD prevalence in China. For instance, Li et al. [5] estimated that the number of individuals with ADRD in China would grow from 16.25 million in 2020 to approximately 48.98 million by 2050. Liu et al. [15] projected that the number of older adults with ADRD would increase from 12.1 million in 2020 to 66.3 million by 2050. The estimates from our study align closely with these figures within a 20% margin. In addition, our analysis shows that informal caregiving accounted for 50% to 70% of ADRD-related costs in China in 2018, a proportion expected to rise to 60% to 80% by 2060. This aligns with global findings, as a systematic review suggests that informal care makes up about 60% of dementia-related costs in low- and middle-income countries [26]. However, the one-child policy implemented in the late 1970s and phased out in 2015 may lead to a shortage of adult caregivers for ADRD patients in China between 2030 and 2060 [27]. Given China’s strong cultural emphasis on filial piety and family caregiving [28] and ongoing debates about whether formal care can adequately meet the emotional and psychological needs of dementia patients [29–34], it remains uncertain how economic development might affect the transition to formal care. There is a pressing need for a robust social care system to increase the proportion of individuals with ADRD receiving long-term care services and programs, improve dementia care efficiency, and provide health education and support for both individuals with ADRD and their caregivers.

Our study also provides a detailed provincial analysis of ADRD prevalence and economic burden from 2025 to 2060. This provincial-level approach reveals substantial geographical disparities in ADRD prevalence and associated costs, highlighting the need for region-specific long-term care strategies. Provinces with a higher proportion of older adults, such as Shandong, Sichuan, Jiangsu, Henan, and Guangdong, are projected to experience a pronounced increase in ADRD cases [19]. Economically, the burden of ADRDs varies substantially across provinces, influenced by regional GDP, healthcare infrastructure, and social support systems [35,36]. Provinces with stronger economies may have more resources to address ADRD costs, whereas less developed regions could face substantial financial challenges [6]. These challenges are exacerbated in provinces with higher proportions of older adults and greater ADRD prevalence, particularly when paired with less developed economies. This disparity highlights the importance of tailored policy interventions that consider the unique socioeconomic contexts of each province.

To address the projected rise in ADRDs and the associated economic burden across China, several targeted policy interventions are recommended. First, public health efforts should promote healthy aging by addressing modifiable risk factors, encouraging physical and mental activity, fostering healthy diets, and managing comorbidities to delay or prevent ADRD and cognitive decline [37]. Second, strengthening regional long-term care infrastructure is essential, particularly in provinces with more aging populations. Expanding dementia care facilities and training specialized care personnel will help meet the growing demand. Third, providing financial aid and caregiver training to support informal caregivers can effectively alleviate family burdens, especially in rural areas with limited access to formal care services. Fourth, provinces with high projected ADRD burdens should integrate ADRD-related benefits into retirement schemes and ensure that resources are allocated to follow individuals across regions, reducing out-of-pocket costs and promoting equitable access to care. Finally, investment in dementia treatment, caregiving research, and public–private partnerships is essential to develop a sustainable care framework addressing the diverse needs of urban and rural populations in China.

This study has several limitations. First, potential underestimation of ADRD cases may have occurred because the CHARLS cognitive and functional assessments serve as proxies for diagnostic criteria. Consistent with prior work [6,7,12], we defined CI as impairment in ≥2 domains using education-adjusted cutoffs. However, the CHARLS cognitive assessments in the analytic waves include only 3 domains (time orientation, memory, and arithmetic) and lack language and broader executive-function tasks. Under the ≥2-domain rule, this limited coverage likely reduces sensitivity, so our ADRD prevalence and cost projections should be interpreted as conservative (lower-bound) estimates. Nonetheless, CHARLS remains the most comprehensive, nationally representative dataset on aging in China, providing a unique opportunity to estimate the burden of aging-related diseases. Second, our ADRD probability model did not include lifestyle factors because these measures were not consistently available across CHARLS waves. This omission may result in residual confounding and limit risk adjustment. Third, formal care costs may have been underestimated because CHARLS provides limited data on social services, such as nursing home care and community-based care. Fourth, data on healthcare costs are self-reported, which may lead to recall bias. Fifth, our projections did not incorporate regional variations in healthcare access, potentially biasing estimates in certain areas, especially rural regions with limited diagnostic capabilities. In addition, we applied a uniform historical growth rate of ADRD prevalence across provinces due to data limitations. These estimates should therefore be viewed as conservative, baseline projections that may evolve with future demographic, policy, or medical changes. Lastly, this study does not consider the potential effects of preventative interventions or treatment breakthroughs, which could reduce disease prevalence or delay onset. This limitation highlights the need for ongoing model adjustments as new clinical and policy developments arise.

## Conclusion

This study projects the prevalence and economic burden of ADRD in China from 2025 to 2060, revealing an alarming increase in cases and associated costs. The findings demonstrate pronounced geographic disparities in ADRD prevalence and economic costs, emphasizing the need for regionally tailored interventions. Investment in healthcare infrastructure, early diagnosis, and dementia-specific education is crucial to mitigate the socioeconomic impact of ADRD. Additionally, strengthening formal care services and offering support to informal caregivers are essential strategies for addressing these challenges. These findings could help policymakers take proactive measures to ensure equitable and sustainable ADRD care across China.

## Data Availability

The CHARLS data are publicly available from the CHARLS website https://charls.pku.edu.cn/. The analytic code is available from the corresponding authors upon request.
