# Supplementary material for: Projected Prevalence and Economic Burden of Alzheimer’s Disease and Related Dementias in China: Regional Disparities and Policy Implications
Source: Health Data Sci. 2025 Nov 21;5:0377. doi: 10.34133/hds.0377 (PMC12635023; doi:10.34133/hds.0377)
Supplement: Supplementary 1 — Figs. S1 and S2 Tables S1 to S16 [file hds.0377.f1.docx]

**Supplemental Materials**

**Contents**

[1. Data Sources 2](#_Toc210141937)

[2. Detailed information of cognitive batteries applied in CHARLS 3](#_Toc210141938)

[3. Demographic and Health Profiles of Sample Population 4](#_Toc210141939)

[4. Probit Regression Results for ADRD Prediction Model 5](#_Toc210141940)

[5. Cost Analysis 6](#_Toc210141941)

[(1) Formal Care Costs 6](#_Toc210141942)

[(2) Informal Care Costs 15](#_Toc210141943)

[(3) Total Medical Costs 19](#_Toc210141944)

[6. Provincial Predictions of ADRD Prevalence and Economic Burden 19](#_Toc210141945)

[7. One-Way Sensitivity Analysis of ADRD Prevalence Growth Rate 21](#_Toc210141946)

# 1. Data Sources

The Table S1 provides a detailed summary of all data sources used in this study, along with their main characteristics. It includes information such as reference details, population represented, data collection methods, collection years, demographic specifics, and sample size, as applicable.

**Table S1. Data Sources and Key Characteristics for ADRD Projections in this study**

| **Data Source** | **Reference/Contact Information** | **Population Represented** | **Data Collection Method** | **Year(s) of Data Collection** | **Sex and Age Range** | **Sample Size** |
| --- | --- | --- | --- | --- | --- | --- |
| China Health and Retirement Longitudinal Study (CHARLS) | CHARLS Team, Peking University (https://charls.pku.edu.cn/) | Nationally representative sample of individuals aged 45 and older in China | Longitudinal household survey via interviews and cognitive/functional assessments | 2011, 2013, 2015, 2018 | Male and female, aged ≥45 | ~17,000 individuals from ~10,000 households |
| China Statistical Yearbook | National Bureau of Statistics of China (http://www.stats.gov.cn/) | National and provincial economic data across China | Governmental statistical data collection | Annual (various years) | Not applicable | Not applicable |
| Provincial Statistical Yearbooks | Respective provincial statistical bureaus | Economic and demographic data for provinces in China | Governmental statistical data collection | Annual (various years) | Not applicable | Not applicable |
| Global Burden of Disease (GBD) Study | Institute for Health Metrics and Evaluation (IHME) (https://www.healthdata.org/) | Global population estimates for ADRD prevalence and trends | Systematic analysis of global health data | 1990–2021 | Male and female, all ages | Not applicable |
| China Alzheimer Report 2024 | Gang W, Jinlei Q, Xinya L et al. China Alzheimer Report 2024. Journal of Diagnostics Concepts & Practice 2024;23(3):219-256. | Nationally representative estimates of ADRD prevalence in China | Compilation of clinical and demographic data | 2021 | Male and female, all ages | Not applicable |
| United Nations World Population Prospects | United Nations Population Division (https://population.un.org/) | Population projections for China and provinces | Statistical modeling and population projections | Medium Variant: 2025–2060 | Male and female, all ages | Not applicable |

# 2. Detailed information of cognitive batteries applied in CHARLS

**Table S2. Detailed Information of Cognitive Batteries Applied in CHARLS**

| Domain assessed in CHARLS | Evaluation process (interviewer prompts) | Scoring criterion | Score range |
| --- | --- | --- | --- |
| Time orientation (TICS items) | Name current year, month, day, day-of-week, season. | 1 point per correct item. | 0–5. |
| Arithmetic / attention (Serial 7s) | Subtract 7 from 100, up to five consecutive subtractions. | 1 point per correct subtraction. | 0–5. |
| Immediate word recall | Immediately recall a list of 10 Chinese nouns. | Number of correct words. | 0–10. |
| Delayed word recall (~4–5 min) | Recall the same 10-word list after a brief interval. | Number of correct words. | 0–10. |
| Episodic memory (composite) | Constructed from immediate & delayed recall. | Average of the two recalls (widely used in CHARLS); equivalently，0.5 × (immediate + delayed). | 0–10. |

# 3. Demographic and Health Profiles of Sample Population

Table S3 presents the basic characteristics of respondents aged 50 and above from the CHARLS database used in this study.

Missing covariates were handled as follows. For time-invariant covariates (e.g., ethnicity, educational level), if a value was missing in a given wave but had been observed previously for the same participant, we carried forward the most recent observed value; if a value was never observed for a participant, no imputation was performed. For time-varying covariates, we did not impute missing values; analyses used available cases, and sample sizes vary accordingly.

**Table S3. Demographic and Health Profiles of CHARLS Respondents Aged 50 and Above**

| **Characteristic** | **Number of respondents** | **Distribution percent** |
| --- | --- | --- |
| Total | 23,891 | 100.00 |
| Gender |  |  |
| Female | 12,132 | 50.78 |
| Male | 11,759 | 49.22 |
| Age |  |  |
| 50–54 yr | 4,004 | 16.75 |
| 55–59 yr | 4,419 | 18.49 |
| 60–64 yr | 3,672 | 15.37 |
| 65–69 yr | 4,148 | 17.36 |
| 70–74 yr | 3,080 | 12.89 |
| 75–79 yr | 2,143 | 8.97 |
| 80–84 yr | 1,312 | 5.49 |
| 85–89 yr | 655 | 2.74 |
| ≥90 yr | 465 | 1.95 |
| Ethnicities *^a^* |  |  |
| Han nationality | 17,148 | 92.22 |
| Others | 1,446 | 7.78 |
| Educational level *^b^* |  |  |
| No formal education | 9,455 | 39.61 |
| Primary school | 5,547 | 23.24 |
| Middle school | 5,359 | 22.45 |
| High school or above | 3,511 | 14.71 |
| Marital status *^c^* |  |  |
| Married | 21,093 | 88.35 |
| Separated/Divorced/Widowed/Never married | 2,782 | 11.65 |
| Household registration *^d^* |  |  |
| Urban | 5,697 | 24.03 |
| Rural | 18,007 | 75.97 |
| Limitations in ADLs *^e^* |  |  |
| No | 14,674 | 61.77 |
| Yes | 9,080 | 38.23 |
| Limitations in IADLs *^f^* |  |  |
| No | 13,433 | 56.53 |
| Yes | 10,330 | 43.47 |
| Self assessed memory level |  |  |
| Poor | 3,309 | 14.51 |
| Fair | 12,489 | 54.78 |
| Good | 3,988 | 17.49 |
| Very good | 2,590 | 11.36 |
| Excellent | 422 | 1.85 |
| Coexisting conditions |  |  |
| Hypertension *^g^* | 10,515 | 44.65 |
| Dyslipidemia *^h^* | 6,809 | 28.98 |
| Diabetes or high blood sugar *^i^* | 3,982 | 16.93 |
| Cancer *^j^* | 832 | 3.54 |
| Chronic lung diseases *^k^* | 4,804 | 20.41 |
| Liver disease *^l^* | 2,292 | 9.74 |
| Heart attack, coronary heart disease, angina, congestive heart failure, or other heart problems *^m^* | 5,940 | 25.23 |
| Stroke *^n^* | 2,212 | 9.39 |
| Kidney disease *^o^* | 3,277 | 13.93 |
| Stomach or other digestive disease *^p^* | 8,524 | 36.21 |
| Emotional, nervous, or psychiatric problems *^q^* | 1,047 | 4.45 |
| Arthritis or rheumatis *^r^* | 11,032 | 46.86 |
| Asthma *^s^* | 1,967 | 8.36 |

Note: Data are based on a total of 23,891 person-years. Percentages of missing data are indicated as follows: a. 690 missing values (2.89%) for Ethnicities; b. 17 missing values (0.07%) for education level; c. 4 missing values (0.02%) for marital status; d. 238 missing values (1.00%) for household registration; e. 450 missing values (1.88%) for limitations in ADLs; f. 294 missing values (1.23%) for limitations in IADLs. The symbols g-s indicate that the respective conditions have missing values of 621 (2.60%), 789 (3.30%), 671 (2.81%), 634 (2.65%), 627 (2.62%), 661 (2.77%), 643 (2.69%), 619 (2.59%), 652 (2.73%), 608 (2.54%), 635 (2.66%), 608 (2.54%), and 626 (2.62%).

# 4. Probit Regression Results for ADRD Prediction Model

To estimate the probability of ADRDs among CHARLS respondents, we adapted methods from Hurd et al. (2013) and Nandi et al. (2024). Using 2011-2018 CHARLS data, we built a Probit model to predict ADRDs likelihood based on age, sex, education, limitations in activities of daily living (ADL and IADL), and cognitive function scores. Cognitive function, assessed by CHARLS, was a composite of mental status (e.g., serial 7s, orientation, drawing tasks) and episodic memory (immediate and delayed recall of 10 words), yielding a score from 1 to 21. The model’s coefficients were then used as the probability *P(ADRDs)* of each respondent having ADRDs. The results of the probit regression model were shown in Table S4.

**Table S4.** **Probit Regression Results for ADRD Prediction Model**

| **Variables** | **Coefficient** | **Robust Standard Error** | **Z value** | **P value** | **95% confidence interval** | |
| --- | --- | --- | --- | --- | --- | --- |
| Gender | 0.058 | 0.058 | 1.00 | 0.317 | -0.056 | 0.173 |
| Age group (Reference: 50-54yr) |  |  |  |  |  |  |
| 55–59 yr | 0.187 | 0.371 | 0.50 | 0.614 | -0.540 | 0.914 |
| 60–64 yr | 0.233 | 0.370 | 0.63 | 0.528 | -0.491 | 0.957 |
| 65–69 yr | 0.451 | 0.369 | 1.22 | 0.222 | -0.272 | 1.173 |
| 70–74 yr | 0.646 | 0.369 | 1.75 | 0.080 | -0.077 | 1.369 |
| 75–79 yr | 0.588 | 0.372 | 1.58 | 0.114 | -0.141 | 1.318 |
| 80–84 yr | 0.521 | 0.383 | 1.36 | 0.174 | -0.229 | 1.271 |
| 85–89 yr | 0.314 | 0.439 | 0.72 | 0.475 | -0.546 | 1.174 |
| ≥90 yr | 0.671 | 0.545 | 1.23 | 0.218 | -0.397 | 1.739 |
| Education level (Reference: No formal education) |  |  |  |  |  |  |
| Primary school | 0.275 | 0.074 | 3.73 | 0.000 | 0.130 | 0.419 |
| Middle school | 0.350 | 0.086 | 4.06 | 0.000 | 0.181 | 0.519 |
| High school or above | 0.474 | 0.101 | 4.69 | 0.000 | 0.276 | 0.671 |
| ADL | 0.083 | 0.022 | 3.77 | 0.000 | 0.040 | 0.126 |
| IADL | 0.270 | 0.023 | 11.59 | 0.000 | 0.224 | 0.315 |
| Scores on cognitive function（last period） | -0.035 | 0.011 | -3.30 | 0.001 | -0.056 | -0.014 |
| Changes in scores on cognitive function (two preceding period) | 0.017 | 0.011 | 1.64 | 0.100 | -0.003 | 0.038 |
| Changes in ADL limitations (two preceding period) | 0.009 | 0.025 | 0.35 | 0.727 | -0.041 | 0.058 |
| Changes in IADL limitations (two preceding period) | 0.046 | 0.025 | 1.80 | 0.071 | -0.004 | 0.095 |
| Constant | -2.686 | 0.381 | -7.05 | 0.000 | -3.432 | -1.939 |

Based on this assignment result of this model (Table S5), the within-sample fit was good, 89.59% of cases were correctly classified.

**Table S5. Model Prediction Results**

| **Dementia** | **Predicted dementia** | | **Total** |
| --- | --- | --- | --- |
|  | **0** | **1** |  |
| 0 | 19,850 | 2,206 | 22,056 |
| 1 | 119 | 170 | 289 |
| Total | 19,969 | 2,376 | 22,345 |

# 5. Cost Analysis

## (1) Formal Care Costs

**Table S6. Analysis of Outpatient Costs Attributable to ADRDs in Formal Care Costs**

| **Variables** | **Outpatient care cost-Unadjusted** | **Outpatient care cost-Adjusted** |
| --- | --- | --- |
| P(ADRDs) | 8,234.119*** | 3,496.855*** |
|  | (3,027.978) | (1,153.240) |
| Wave (Reference: wave 3) | -247.861*** | -60.083 |
|  | (95.874) | (80.590) |
| Sex (Reference: female) |  | 61.337 |
|  |  | (100.022) |
| Age (Reference: 50-54yr) |  |  |
| 55–59 yr |  | -229.489 |
|  |  | (220.049) |
| 60–64 yr |  | -48.169 |
|  |  | (201.757) |
| 65–69 yr |  | -185.776 |
|  |  | (143.094) |
| 70–74 yr |  | -167.984 |
|  |  | (145.594) |
| 75–79 yr |  | -238.148 |
|  |  | (175.696) |
| 80–84 yr |  | -443.981** |
|  |  | (201.290) |
| 85–89 yr |  | -24.486 |
|  |  | (348.387) |
| ≥90 yr |  | -527.249*** |
|  |  | (182.481) |
| Nation (Reference: Others) |  | 143.785 |
|  |  | (89.802) |
| Educational level (Reference: No formal education) |  |  |
| Primary school |  | -76.189 |
|  |  | (88.122) |
| Middle school |  | 29.616 |
|  |  | (126.601) |
| High school graduate or above |  | 51.087 |
|  |  | (139.124) |
| Marital status (Reference: Separated/Divorced/Widowed/Never married) |  | 97.201 |
|  |  | (86.071) |
| Household registration (Reference: Urban) |  | -120.408 |
|  |  | (107.618) |
| Area (Reference: Eastern region) |  |  |
| Central Region |  | 247.928* |
|  |  | (138.203) |
| Western region |  | 72.191 |
|  |  | (72.570) |
| Northeastern region |  | 105.340 |
|  |  | (129.928) |
| Number of children |  | 7.110 |
|  |  | (45.099) |
| Annual per capita household consumption |  | 0.006** |
|  |  | (0.003) |
| Coexisting conditions |  |  |
| hypertension |  | 56.631 |
|  |  | (82.167) |
| dyslipidemia |  | 225.087** |
|  |  | (114.489) |
| diabetes |  | -42.606 |
|  |  | (126.957) |
| cancer |  | 2,855.039* |
|  |  | (1,517.337) |
| chronic lung diseases |  | 95.323 |
|  |  | (116.733) |
| liver disease |  | 13.252 |
|  |  | (172.300) |
| cardiovascular conditions |  | 327.636*** |
|  |  | (109.769) |
| stroke |  | 71.642 |
|  |  | (179.767) |
| kidney disease |  | 112.373 |
|  |  | (144.846) |
| gastrointestinal disorders |  | 69.282 |
|  |  | (87.193) |
| psychiatric conditions |  | -243.950** |
|  |  | (122.594) |
| arthritis or rheumatism |  | 104.915 |
|  |  | (77.108) |
| asthma |  | 95.123 |
|  |  | (153.001) |
| Constant | 583.939*** | -68.589 |
|  | (64.779) | (346.692) |
| Observations | 14,582 | 10,262 |
| R-squared | 0.005 | 0.017 |

Robust standard errors in parentheses

*** p<0.01, ** p<0.05, * p<0.1

**Table S7. Analysis of Hospitalization Cost Attributable to ADRDs in Formal Care Costs**

| **Variables** | **Hospitalization cost-Unadjusted** | **Hospitalization cost-Adjusted** |
| --- | --- | --- |
| P(ADRDs) | 6,279.313*** | 4,830.535*** |
|  | (967.524) | (1,290.316) |
| Wave (Reference: wave 3) | 77.154** | -0.711 |
|  | (35.153) | (39.148) |
| Sex (Reference: female) |  | 131.951*** |
|  |  | (48.785) |
| Age (Reference: 50-54yr) |  |  |
| 55–59 yr |  | -136.765 |
|  |  | (84.255) |
| 60–64 yr |  | -10.424 |
|  |  | (67.899) |
| 65–69 yr |  | -29.591 |
|  |  | (63.880) |
| 70–74 yr |  | 46.238 |
|  |  | (80.326) |
| 75–79 yr |  | -69.238 |
|  |  | (98.380) |
| 80–84 yr |  | 118.731 |
|  |  | (233.122) |
| 85–89 yr |  | -71.023 |
|  |  | (276.376) |
| ≥90 yr |  | -45.326 |
|  |  | (237.809) |
| Nation (Reference: Others) |  | 196.504*** |
|  |  | (57.038) |
| Educational level (Reference: No formal education) |  |  |
| Primary school |  | -40.496 |
|  |  | (54.260) |
| Middle school |  | -5.731 |
|  |  | (59.763) |
| High school graduate or above |  | -46.754 |
|  |  | (86.110) |
| Marital status (Reference: Separated/Divorced/Widowed/Never married) |  | 36.574 |
|  |  | (60.825) |
| Household registration (Reference: Urban) |  | -178.288*** |
|  |  | (52.426) |
| Area (Reference: Eastern region) |  |  |
| Central Region |  | -25.132 |
|  |  | (55.090) |
| Western region |  | -19.348 |
|  |  | (52.433) |
| Northeastern region |  | -9.967 |
|  |  | (126.608) |
| Number of children |  | 37.796 |
|  |  | (22.993) |
| Annual per capita household consumption |  | 0.011*** |
|  |  | (0.004) |
| Coexisting conditions |  |  |
| hypertension |  | 61.923 |
|  |  | (44.618) |
| dyslipidemia |  | 89.069 |
|  |  | (63.294) |
| diabetes |  | 31.387 |
|  |  | (80.386) |
| cancer |  | 2,799.309*** |
|  |  | (661.160) |
| chronic lung diseases |  | 171.789** |
|  |  | (86.885) |
| liver disease |  | 172.660 |
|  |  | (138.841) |
| cardiovascular conditions |  | 279.892*** |
|  |  | (59.643) |
| stroke |  | 38.797 |
|  |  | (108.171) |
| kidney disease |  | 342.251*** |
|  |  | (107.150) |
| gastrointestinal disorders |  | 5.870 |
|  |  | (53.820) |
| psychiatric conditions |  | -269.750*** |
|  |  | (91.585) |
| arthritis or rheumatism |  | -69.656 |
|  |  | (45.676) |
| asthma |  | 129.848 |
|  |  | (131.207) |
| Constant | 251.609*** | -309.124* |
|  | (27.023) | (162.787) |
| Observations | 14,565 | 10,258 |
| R-squared | 0.014 | 0.078 |

Robust standard errors in parentheses

*** p<0.01, ** p<0.05, * p<0.1

**Table S8. Analysis of OOP Cost Attributable to ADRDs in Formal Care Costs**

| **Variables** | **Total out-of-pocket spending-Unadjusted** | **Total out-of-pocket spending-Adjusted** |
| --- | --- | --- |
| P(ADRDs) | 8,500.804*** | 4,553.338*** |
|  | (2,136.838) | (972.307) |
| Wave (Reference: wave 3) | -177.021*** | -66.787 |
|  | (67.003) | (59.038) |
| Sex (Reference: female) |  | 63.917 |
|  |  | (67.168) |
| Age (Reference: 50-54yr) |  |  |
| 55–59 yr |  | -119.871 |
|  |  | (143.367) |
| 60–64 yr |  | 49.793 |
|  |  | (125.065) |
| 65–69 yr |  | 0.395 |
|  |  | (106.057) |
| 70–74 yr |  | -84.679 |
|  |  | (105.625) |
| 75–79 yr |  | -85.788 |
|  |  | (136.898) |
| 80–84 yr |  | -327.637** |
|  |  | (140.117) |
| 85–89 yr |  | -5.094 |
|  |  | (265.861) |
| ≥90 yr |  | -328.080** |
|  |  | (167.232) |
| Nation (Reference: Others) |  | 180.523*** |
|  |  | (67.526) |
| Educational level (Reference: No formal education) |  |  |
| Primary school |  | -16.792 |
|  |  | (67.244) |
| Middle school |  | -11.356 |
|  |  | (85.186) |
| High school graduate or above |  | -50.830 |
|  |  | (101.362) |
| Marital status (Reference: Separated/Divorced/Widowed/Never married) |  | 137.884** |
|  |  | (59.945) |
| Household registration (Reference: Urban) |  | -141.356* |
|  |  | (82.825) |
| Area (Reference: Eastern region) |  |  |
| Central Region |  | 80.652 |
|  |  | (88.756) |
| Western region |  | 1.305 |
|  |  | (57.393) |
| Northeastern region |  | 106.803 |
|  |  | (109.910) |
| Number of children |  | 39.203 |
|  |  | (29.433) |
| Annual per capita household consumption |  | 0.009*** |
|  |  | (0.003) |
| Coexisting conditions |  |  |
| hypertension |  | 50.835 |
|  |  | (58.921) |
| dyslipidemia |  | 146.592* |
|  |  | (79.329) |
| diabetes |  | -40.771 |
|  |  | (87.186) |
| cancer |  | 2,982.353*** |
|  |  | (910.542) |
| chronic lung diseases |  | 198.848** |
|  |  | (97.597) |
| liver disease |  | 72.922 |
|  |  | (120.302) |
| cardiovascular conditions |  | 337.604*** |
|  |  | (80.295) |
| stroke |  | 101.682 |
|  |  | (155.330) |
| kidney disease |  | 115.961 |
|  |  | (94.154) |
| gastrointestinal disorders |  | 71.012 |
|  |  | (64.198) |
| psychiatric conditions |  | -125.303 |
|  |  | (115.334) |
| arthritis or rheumatism |  | 66.451 |
|  |  | (57.020) |
| asthma |  | 55.649 |
|  |  | (119.855) |
| Constant | 524.997*** | -288.712 |
|  | (47.989) | (226.509) |
| Observations | 14,371 | 10,139 |
| R-squared | 0.010 | 0.042 |

Robust standard errors in parentheses

*** p<0.01, ** p<0.05, * p<0.1

**Table S9. Analysis of Total Medical Cost Attributable to ADRDs in Formal Care Costs**

| **Variables** | **Total medical cost-Unadjusted** | **Total medical cost-Adjusted** |
| --- | --- | --- |
| P(ADRDs) | 14,819.325*** | 8,288.182*** |
|  | (3,284.796) | (1,703.077) |
| Wave (Reference: wave 3) | -166.085 | -55.192 |
|  | (106.344) | (94.834) |
| Sex (Reference: female) |  | 198.305* |
|  |  | (117.034) |
| Age (Reference: 50-54yr) |  |  |
| 55–59 yr |  | -361.667 |
|  |  | (243.202) |
| 60–64 yr |  | -61.302 |
|  |  | (225.876) |
| 65–69 yr |  | -215.212 |
|  |  | (163.363) |
| 70–74 yr |  | -135.410 |
|  |  | (170.176) |
| 75–79 yr |  | -303.342 |
|  |  | (207.487) |
| 80–84 yr |  | -302.177 |
|  |  | (310.689) |
| 85–89 yr |  | -84.976 |
|  |  | (446.118) |
| ≥90 yr |  | -573.026** |
|  |  | (291.207) |
| Nation (Reference: Others) |  | 339.478*** |
|  |  | (117.063) |
| Educational level (Reference: No formal education) |  |  |
| Primary school |  | -113.784 |
|  |  | (106.346) |
| Middle school |  | 24.734 |
|  |  | (146.617) |
| High school graduate or above |  | 0.075 |
|  |  | (169.820) |
| Marital status (Reference: Separated/Divorced/Widowed/Never married) |  | 129.734 |
|  |  | (109.501) |
| Household registration (Reference: Urban) |  | -303.544** |
|  |  | (123.881) |
| Area (Reference: Eastern region) |  |  |
| Central Region |  | 217.004 |
|  |  | (157.129) |
| Western region |  | 51.432 |
|  |  | (94.491) |
| Northeastern region |  | 94.305 |
|  |  | (181.651) |
| Number of children |  | 45.508 |
|  |  | (53.240) |
| Annual per capita household consumption |  | 0.017*** |
|  |  | (0.006) |
| Coexisting conditions |  |  |
| hypertension |  | 113.158 |
|  |  | (99.238) |
| dyslipidemia |  | 302.896** |
|  |  | (136.241) |
| diabetes |  | -0.086 |
|  |  | (156.879) |
| cancer |  | 5,730.642*** |
|  |  | (1,772.668) |
| chronic lung diseases |  | 275.275* |
|  |  | (149.119) |
| liver disease |  | 194.664 |
|  |  | (233.415) |
| cardiovascular conditions |  | 625.552*** |
|  |  | (130.147) |
| stroke |  | 126.869 |
|  |  | (213.461) |
| kidney disease |  | 441.995** |
|  |  | (187.859) |
| gastrointestinal disorders |  | 70.145 |
|  |  | (107.434) |
| psychiatric conditions |  | -523.954*** |
|  |  | (165.684) |
| arthritis or rheumatism |  | 37.962 |
|  |  | (93.867) |
| asthma |  | 231.487 |
|  |  | (209.831) |
| Constant | 829.887*** | -375.153 |
|  | (73.484) | (409.586) |
| Observations | 14,432 | 10,192 |
| R-squared | 0.011 | 0.051 |

Robust standard errors in parentheses

*** p<0.01, ** p<0.05, * p<0.1

**Table S10. Analysis of Formal Care Cost through 2,000 bootstrap simulations**

| **Variables** | **Outpatient care cost-Unadjusted** | **Outpatient care cost-Adjusted** | **Hospitalization cost-Unadjusted** | **Hospitalization cost-Adjusted** | **Total out-of-pocket spending-Unadjusted** | **Total out-of-pocket spending-Adjusted** | **Total medical cost-Unadjusted** | **Total medical cost-Adjusted** |
| --- | --- | --- | --- | --- | --- | --- | --- | --- |
| P(ADRDs) | 8,234.119*** | 3,496.855*** | 6,279.313*** | 4,830.535*** | 8,500.804*** | 4,553.338*** | 14,819.325*** | 8,288.182*** |
|  | (2,262.156 - 14,206.082) | (1,187.191 - 5,806.519) | (4,456.865 - 8,101.761) | (2,332.293 - 7,328.778) | (4,300.341 - 12,701.267) | (2,574.613 - 6,532.064) | (8,427.645 - 21,211.006) | (4,838.148 - 11,738.216) |
| Constant | 583.939*** | -68.589 | 251.609*** | -309.124* | 524.997*** | -288.712 | 829.887*** | -375.153 |
|  | (454.765 - 713.113) | (-739.063 - 601.885) | (198.248 - 304.970) | (-630.676 - 12.428) | (429.757 - 620.236) | (-719.169 - 141.746) | (688.353 - 971.420) | (-1,163.232 - 412.926) |
| Observations | 14,582 | 10,262 | 14,565 | 10,258 | 14,371 | 10,139 | 14,432 | 10,192 |

95% CI in parentheses

*** p<0.01, ** p<0.05, * p<0.1

## (2) Informal Care Costs

**Table S11. Analysis of Informal Care Costs Attributable to ADRDs Based on Salary of Service Industry**

| **Variables** | **Caregiving time valued according to replacement cost of service industry-Unadjusted** | **Caregiving time valued according to replacement cost of service industry-Adjusted** |
| --- | --- | --- |
| P(ADRDs) | 18,859.616*** | 17,047.786*** |
|  | (828.907) | (1,050.228) |
| Wave (Reference: wave 3) | -39.292 | 25.651 |
|  | (28.695) | (32.777) |
| Sex (Reference: female) |  | -163.564*** |
|  |  | (36.097) |
| Age (Reference: 50-54yr) |  |  |
| 55–59 yr |  | 575.158*** |
|  |  | (161.996) |
| 60–64 yr |  | 278.379*** |
|  |  | (56.676) |
| 65–69 yr |  | 304.275*** |
|  |  | (58.274) |
| 70–74 yr |  | 211.005*** |
|  |  | (59.294) |
| 75–79 yr |  | 151.667* |
|  |  | (88.425) |
| 80–84 yr |  | 368.393** |
|  |  | (158.661) |
| 85–89 yr |  | 1,407.337*** |
|  |  | (483.338) |
| ≥90 yr |  | 714.409 |
|  |  | (789.396) |
| Nation (Reference: Others) |  | -101.166 |
|  |  | (80.192) |
| Educational level (Reference: No formal education) |  |  |
| Primary school |  | -172.569*** |
|  |  | (50.498) |
| Middle school |  | -290.990*** |
|  |  | (43.762) |
| High school graduate or above |  | -373.930*** |
|  |  | (48.211) |
| Marital status (Reference: Separated/Divorced/Widowed/Never married) |  | -113.344* |
|  |  | (66.048) |
| Household registration (Reference: Urban) |  | -24.097 |
|  |  | (41.063) |
| Area (Reference: Eastern region) |  |  |
| Central Region |  | -24.052 |
|  |  | (41.207) |
| Western region |  | 42.186 |
|  |  | (41.167) |
| Northeastern region |  | 73.800 |
|  |  | (69.678) |
| Number of children |  | 71.787*** |
|  |  | (17.077) |
| Annual per capita household consumption |  | 0.000 |
|  |  | (0.001) |
| Coexisting conditions |  |  |
| hypertension |  | 52.635 |
|  |  | (36.508) |
| dyslipidemia |  | 24.824 |
|  |  | (43.856) |
| diabetes |  | 8.774 |
|  |  | (51.089) |
| cancer |  | 268.608 |
|  |  | (168.958) |
| chronic lung diseases |  | 26.437 |
|  |  | (52.632) |
| liver disease |  | 9.780 |
|  |  | (72.238) |
| cardiovascular conditions |  | 91.616* |
|  |  | (48.046) |
| stroke |  | 158.892* |
|  |  | (92.514) |
| kidney disease |  | 27.077 |
|  |  | (62.481) |
| gastrointestinal disorders |  | 10.507 |
|  |  | (39.266) |
| psychiatric conditions |  | 329.695** |
|  |  | (162.341) |
| arthritis or rheumatism |  | 15.188 |
|  |  | (35.673) |
| asthma |  | 193.460** |
|  |  | (97.634) |
| Constant | 214.641*** | 69.745 |
|  | (22.862) | (131.648) |
| Observations | 14,731 | 10,336 |
| R-squared | 0.169 | 0.185 |

Robust standard errors in parentheses

*** p<0.01, ** p<0.05, * p<0.1

**Table S12. Analysis of Informal Care Costs Attributable to ADRDs Based on Salary of Healthcare Industry**

| **Variables** | **Caregiving time valued according to replacement cost of healthcare industry-Unadjusted** | **Caregiving time valued according to replacement cost of healthcare industry-Adjusted** |
| --- | --- | --- |
| P(ADRDs) | 32,386.742*** | 29,920.975*** |
|  | (1,446.056) | (1,896.036) |
| Wave (Reference: wave 3) | 24.923 | 93.969 |
|  | (50.531) | (58.542) |
| Sex (Reference: female) |  | -272.618*** |
|  |  | (65.459) |
| Age (Reference: 50-54yr) |  |  |
| 55–59 yr |  | 918.635*** |
|  |  | (260.845) |
| 60–64 yr |  | 447.204*** |
|  |  | (106.306) |
| 65–69 yr |  | 492.601*** |
|  |  | (109.818) |
| 70–74 yr |  | 312.772*** |
|  |  | (110.719) |
| 75–79 yr |  | 282.176* |
|  |  | (165.684) |
| 80–84 yr |  | 687.086** |
|  |  | (298.017) |
| 85–89 yr |  | 2,424.213*** |
|  |  | (854.353) |
| ≥90 yr |  | 1,316.824 |
|  |  | (1,470.785) |
| Nation (Reference: Others) |  | -147.372 |
|  |  | (142.458) |
| Educational level (Reference: No formal education) |  |  |
| Primary school |  | -359.428*** |
|  |  | (92.695) |
| Middle school |  | -573.041*** |
|  |  | (78.071) |
| High school graduate or above |  | -704.248*** |
|  |  | (87.646) |
| Marital status (Reference: Separated/Divorced/Widowed/Never married) |  | -160.126 |
|  |  | (118.537) |
| Household registration (Reference: Urban) |  | -22.919 |
|  |  | (73.722) |
| Area (Reference: Eastern region) |  |  |
| Central Region |  | -124.578* |
|  |  | (75.591) |
| Western region |  | 100.617 |
|  |  | (78.631) |
| Northeastern region |  | -74.754 |
|  |  | (108.748) |
| Number of children |  | 119.221*** |
|  |  | (31.120) |
| Annual per capita household consumption |  | 0.001 |
|  |  | (0.001) |
| Coexisting conditions |  |  |
| hypertension |  | 90.208 |
|  |  | (66.273) |
| dyslipidemia |  | 43.793 |
|  |  | (78.685) |
| diabetes |  | 22.391 |
|  |  | (94.031) |
| cancer |  | 537.582* |
|  |  | (307.723) |
| chronic lung diseases |  | 75.184 |
|  |  | (98.749) |
| liver disease |  | -21.435 |
|  |  | (123.779) |
| cardiovascular conditions |  | 156.254* |
|  |  | (87.486) |
| stroke |  | 271.063 |
|  |  | (168.722) |
| kidney disease |  | 27.995 |
|  |  | (112.401) |
| gastrointestinal disorders |  | -2.653 |
|  |  | (71.666) |
| psychiatric conditions |  | 709.442** |
|  |  | (313.641) |
| arthritis or rheumatism |  | 63.259 |
|  |  | (64.668) |
| asthma |  | 351.233* |
|  |  | (185.047) |
| Constant | 337.393*** | 128.562 |
|  | (39.192) | (238.485) |
| Observations | 14,731 | 10,336 |
| R-squared | 0.157 | 0.176 |

Robust standard errors in parentheses

*** p<0.01, ** p<0.05, * p<0.1

**Table S13. Analysis of Informal Care Cost through 2,000 bootstrap simulations**

| **Variables** | **Caregiving time valued according to replacement cost of service industry-Unadjusted** | **Caregiving time valued according to replacement cost of service industry-Adjusted** | **Caregiving time valued according to replacement cost of healthcare industry-Unadjusted** | **Caregiving time valued according to replacement cost of healthcare industry-Adjusted** |
| --- | --- | --- | --- | --- |
| P(ADRDs) | 18,859.616*** | 17,047.786*** | 32,386.742*** | 29,920.975*** |
|  | (17,293.636 - 20,425.595) | (15,012.479 - 19,083.094) | (29,678.084 - 35,095.399) | (26,245.625 - 33,596.326) |
| Constant | 214.641*** | 69.745 | 337.393*** | 128.562 |
|  | (168.936 - 260.345) | (-185.666 - 325.157) | (259.040 - 415.746) | (-333.796 - 590.920) |
| Observations | 14,731 | 10,336 | 14,731 | 10,336 |

95% CI in parentheses

*** p<0.01, ** p<0.05, * p<0.1

## (3) Total Medical Costs

**Table S14. Analysis of Total Cost through 2000 bootstrap simulations**

| **Variables** | **Care purchased in marketplace plus caregiving time valued according to cost of forgone wages-service industry-Unadjusted** | **Care purchased in marketplace plus caregiving time valued according to cost of forgone wages-service industry-Adjusted** | **Care purchased in marketplace plus caregiving time valued according to cost of forgone wages-healthcare industry-Unadjusted** | **Care purchased in marketplace plus caregiving time valued according to cost of forgone wages-healthcare industry-Adjusted** |
| --- | --- | --- | --- | --- |
| P(ADRDs) | 33,591.715*** | 25,152.611*** | 47,052.796*** | 37,775.629*** |
|  | (26,698.326 - 40,485.105) | (21,025.979 - 29,279.244) | (39,682.355 - 54,423.237) | (32,433.707 - 43,117.552) |
| Constant | 1,032.300*** | -318.738 | 1,144.828*** | -271.651 |
|  | (879.529 - 1,185.071) | (-1,170.402 - 532.926) | (978.925 - 1,310.731) | (-1,207.048 - 663.747) |
| Observations | 14,426 | 10,188 | 14,426 | 10,188 |

CI in parentheses

*** p<0.01, ** p<0.05, * p<0.1

# 6. Provincial Predictions of ADRD Prevalence and Economic Burden

**Table S15. Provincial Predictions of ADRD Prevalence and Economic Burden in China, 2025**

| **Provinces** | **Number of patients with ADRDs** | **Formal Care Cost**  **(billions of 2018 US dollar)** | **Grand Total Cost**  **(billions of 2018 US$)** | |
| --- | --- | --- | --- | --- |
|  |  |  | **Total formal care cost plus caregiving time valued according to replacement cost of service industry** | **Total formal care cost plus caregiving time valued according to replacement cost of healthcare industry** |
| Anhui | 916,912 | 9.35 | 28.36 | 42.60 |
| Beijing | 292,998 | 2.99 | 9.06 | 13.61 |
| Chongqing | 528,747 | 5.39 | 16.36 | 24.57 |
| Fujian | 506,349 | 5.16 | 15.66 | 23.52 |
| Gansu | 309,863 | 3.16 | 9.59 | 14.40 |
| Guangdong | 1,205,213 | 12.29 | 37.28 | 55.99 |
| Guangxi | 655,082 | 6.68 | 20.26 | 30.43 |
| Guizhou | 425,076 | 4.33 | 13.15 | 19.75 |
| Hainan | 121,235 | 1.24 | 3.75 | 5.63 |
| Hebei | 1,035,313 | 10.55 | 32.03 | 48.10 |
| Heilongjiang | 461,440 | 4.70 | 14.27 | 21.44 |
| Henan | 1,399,020 | 14.26 | 43.28 | 65.00 |
| Hubei | 831,515 | 8.48 | 25.72 | 38.63 |
| Hunan | 990,701 | 10.10 | 30.65 | 46.03 |
| Neimenggu | 307,213 | 3.13 | 9.50 | 14.27 |
| Jiangsu | 1,409,316 | 14.37 | 43.60 | 65.48 |
| Jiangxi | 464,462 | 4.73 | 14.37 | 21.58 |
| Jilin | 369,023 | 3.76 | 11.42 | 17.14 |
| Liaoning | 697,751 | 7.11 | 21.58 | 32.42 |
| Ningxia | 66,909 | 0.68 | 2.07 | 3.11 |
| Qinghai | 53,236 | 0.54 | 1.65 | 2.47 |
| Shaanxi | 518,068 | 5.28 | 16.03 | 24.07 |
| Shandong | 1,464,094 | 14.92 | 45.29 | 68.02 |
| Shanghai | 430,685 | 4.39 | 13.32 | 20.01 |
| Shanxi | 395,229 | 4.03 | 12.23 | 18.36 |
| Sichuan | 1,408,669 | 14.36 | 43.58 | 65.45 |
| Tianjin | 194,493 | 1.98 | 6.02 | 9.04 |
| Xizang | 20,861 | 0.21 | 0.65 | 0.97 |
| Xinjiang | 195,385 | 1.99 | 6.04 | 9.08 |
| Yunnan | 539,624 | 5.50 | 16.69 | 25.07 |
| Zhejiang | 888,488 | 9.06 | 27.48 | 41.28 |

**Table S16. Provincial Predictions of ADRD Prevalence and Economic Burden in China, 2060.**

| **Provinces** | **Number of patients with ADRDs** | **Formal Care Cost**  **(billions of 2018 US dollar)** | **Grand Total Cost**  **(billions of 2018 US$)** | |
| --- | --- | --- | --- | --- |
|  |  |  | **Total formal care cost plus caregiving time valued according to replacement cost of service industry** | **Total formal care cost plus caregiving time valued according to replacement cost of healthcare industry** |
| Anhui | 2,225,316 | 63.83 | 193.70 | 290.92 |
| Beijing | 711,096 | 20.40 | 61.90 | 92.96 |
| Chongqing | 1,283,252 | 36.81 | 111.70 | 167.76 |
| Fujian | 1,228,894 | 35.25 | 106.97 | 160.65 |
| Gansu | 752,027 | 21.57 | 65.46 | 98.31 |
| Guangdong | 2,925,014 | 83.90 | 254.61 | 382.39 |
| Guangxi | 1,589,862 | 45.60 | 138.39 | 207.84 |
| Guizhou | 1,031,645 | 29.59 | 89.80 | 134.87 |
| Hainan | 294,232 | 8.44 | 25.61 | 38.46 |
| Hebei | 2,512,672 | 72.07 | 218.72 | 328.48 |
| Heilongjiang | 1,119,900 | 32.12 | 97.48 | 146.40 |
| Henan | 3,395,376 | 97.39 | 295.55 | 443.88 |
| Hubei | 2,018,059 | 57.88 | 175.66 | 263.82 |
| Hunan | 2,404,400 | 68.97 | 209.29 | 314.33 |
| Neimenggu | 745,595 | 21.39 | 64.90 | 97.47 |
| Jiangsu | 3,420,365 | 98.11 | 297.73 | 447.14 |
| Jiangxi | 1,127,235 | 32.33 | 98.12 | 147.36 |
| Jilin | 895,607 | 25.69 | 77.96 | 117.08 |
| Liaoning | 1,693,418 | 48.57 | 147.40 | 221.38 |
| Ningxia | 162,386 | 4.66 | 14.14 | 21.23 |
| Qinghai | 129,201 | 3.71 | 11.25 | 16.89 |
| Shaanxi | 1,257,334 | 36.06 | 109.45 | 164.37 |
| Shandong | 3,553,308 | 101.92 | 309.30 | 464.52 |
| Shanghai | 1,045,259 | 29.98 | 90.99 | 136.65 |
| Shanxi | 959,208 | 27.51 | 83.49 | 125.40 |
| Sichuan | 3,418,794 | 98.06 | 297.59 | 446.94 |
| Tianjin | 472,029 | 13.54 | 41.09 | 61.71 |
| Xizang | 50,629 | 1.45 | 4.41 | 6.62 |
| Xinjiang | 474,193 | 13.60 | 41.28 | 61.99 |
| Yunnan | 1,309,649 | 37.56 | 114.00 | 171.21 |
| Zhejiang | 2,156,331 | 61.85 | 187.70 | 281.90 |

# 7. One-Way Sensitivity Analysis of ADRD Prevalence Growth Rate

Figure S1 and Figure S2 present the results of a one-way sensitivity analysis on the annual growth rate of ADRD prevalence.

Figure S1 illustrates the projected total number of ADRD cases in China by 2060 under different annual prevalence growth rate scenarios. The x-axis represents deviations of ±30% from the baseline growth rate (3.22%), which was derived from the Global Burden of Disease (GBD) Study (1990–2021). The y-axis shows the corresponding projected ADRD prevalence in 2060.


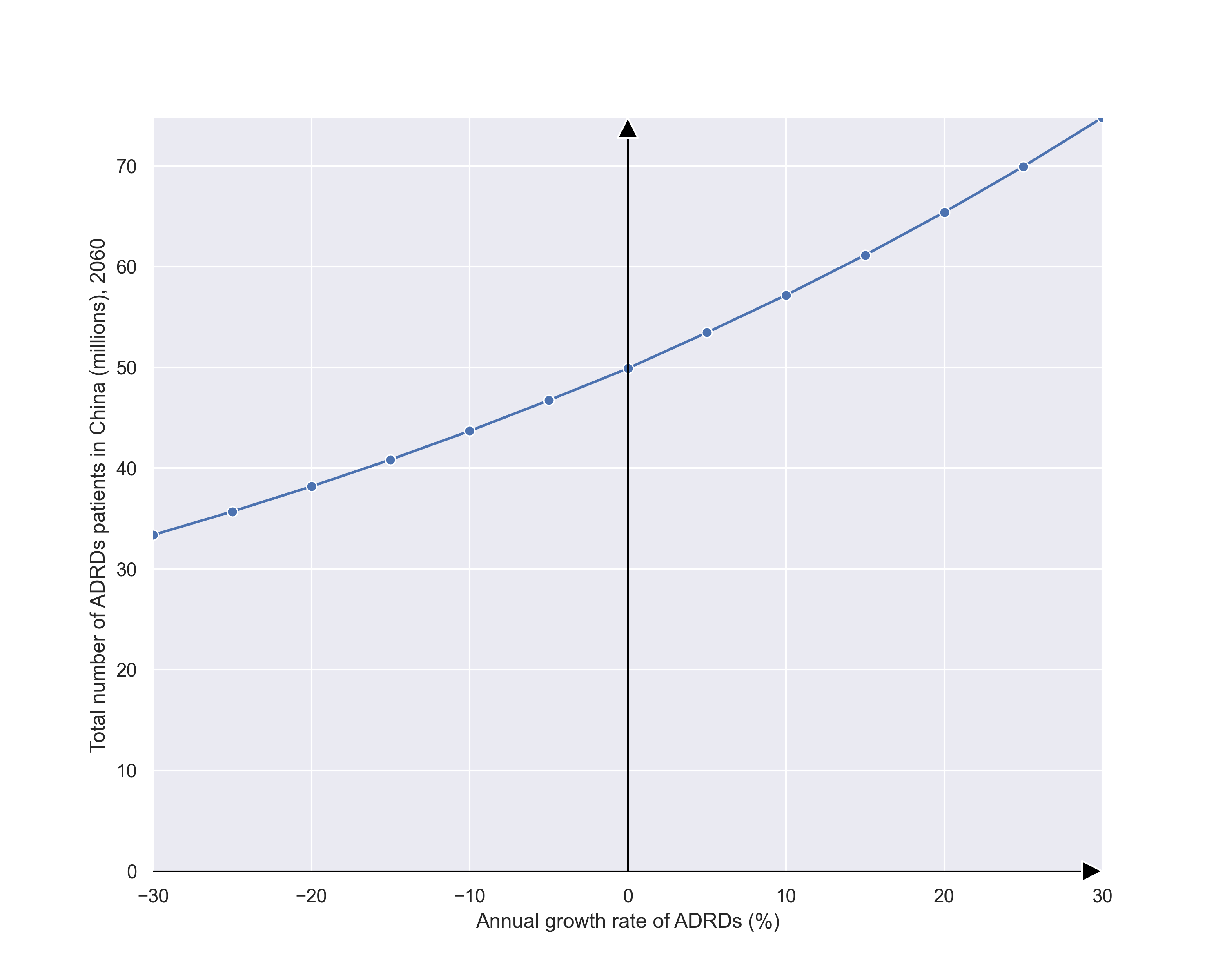


**Figure S1. Projected Total ADRD Cases in China by 2060 Under Varying Annual Prevalence Growth Rates.** The x-axis represents deviations of ±30% from the baseline annual ADRD prevalence growth rate (3.22%, based on the GBD Study 1990–2021), while the y-axis shows the corresponding projected total ADRD cases in China by 2060.

Figure S2 presents the estimated total ADRD-related costs in China by 2060 under the same growth rate variations. The x-axis reflects changes in the annual prevalence growth rate, while the y-axis represents total costs under two valuation approaches: (1) Blue line: Total formal care cost plus caregiving time valued based on the replacement cost of the service industry. (2) Red line: Total formal care cost plus caregiving time valued based on the replacement cost of the healthcare industry.

These results provide insight into the sensitivity of our projections to changes in ADRD prevalence growth assumptions.


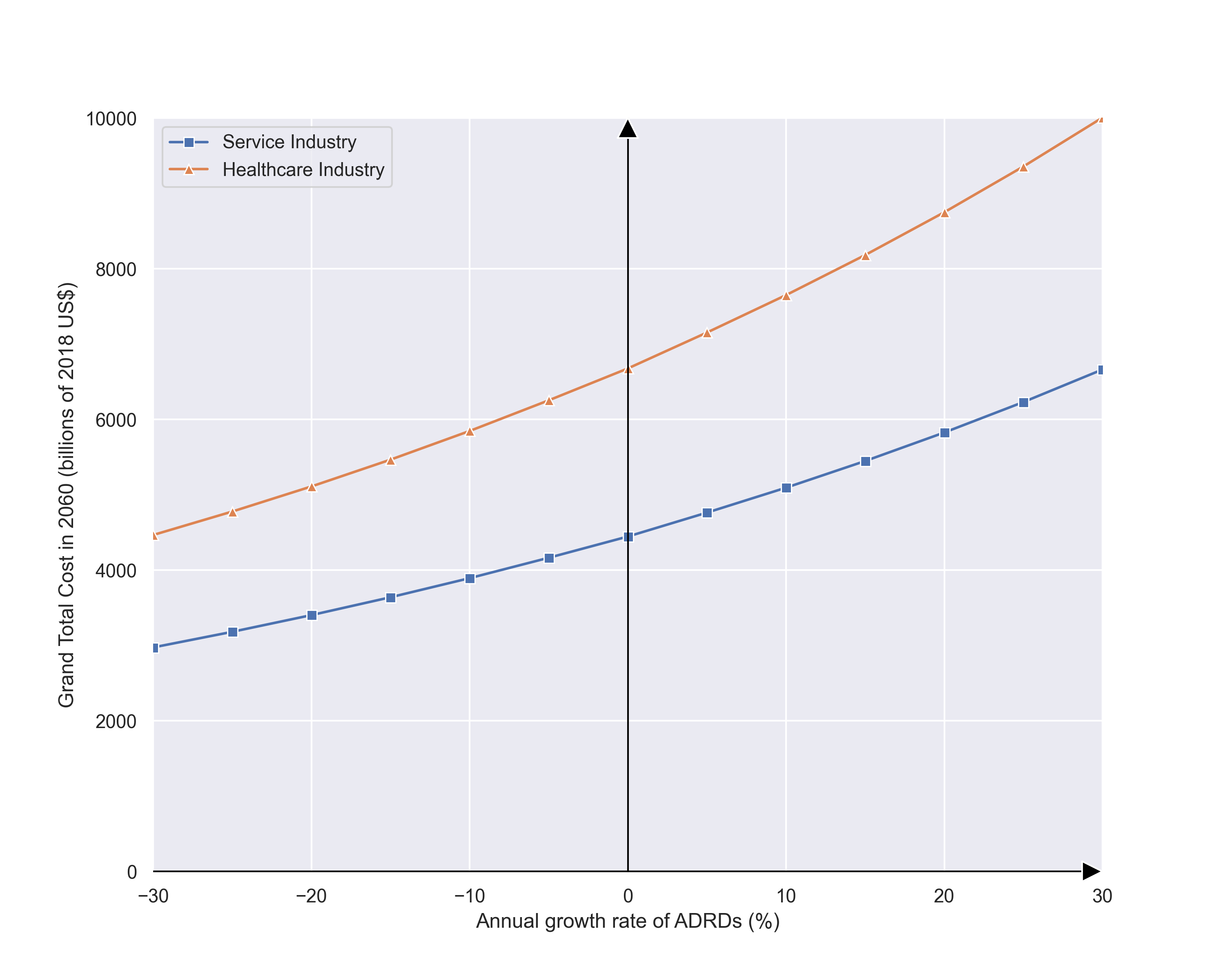


**Figure S2. Projected Total ADRD-Related Costs in China by 2060 Under Varying Annual Prevalence Growth Rates.** The x-axis represents deviations of ±30% from the baseline annual ADRD prevalence growth rate (3.22%, based on the GBD Study 1990–2021), while the y-axis displays the total projected ADRD-related costs under two cost valuation approaches: blue line (replacement cost of the service industry) and red line (replacement cost of the healthcare industry).
